# Fiber Bragg Grating Thermometry and Post-Treatment Ablation Size Analysis of Radiofrequency Thermal Ablation on Ex Vivo Liver, Kidney and Lung

**DOI:** 10.3390/s25010245

**Published:** 2025-01-03

**Authors:** Sanzhar Korganbayev, Leonardo Bianchi, Clara Girgi, Elva Vergantino, Domiziana Santucci, Eliodoro Faiella, Paola Saccomandi

**Affiliations:** 1 Department of Mechanical Engineering, Politecnico di Milano, Via Giuseppe La Masa 1, 20156 Milan, Italy; sanzhar.korganbayev@polimi.it (S.K.); leonardo.bianchi@polimi.it (L.B.); clara.girgi@mail.polimi.it (C.G.); 2 Operative Research Unit of Radiology and Interventional Radiology, Fondazione Policlinico Universitario Campus Bio-Medico, Via Alvaro del Portillo 200, 00128 Rome, Italy; elva.vergantino@unicampus.it (E.V.); d.santucci@policlinicocampus.it (D.S.); e.faiella@policlinicocampus.it (E.F.); 3 Research Unit of Radiology and Interventional Radiology, Department of Medicine and Surgery, Università Campus Bio-Medico di Roma, Via Alvaro del Portillo 21, 00128 Rome, Italy

**Keywords:** fiber Bragg grating sensors, thermometry, radiofrequency ablation, liver, kidney, lung

## Abstract

Radiofrequency ablation (RFA) is a minimally invasive procedure that utilizes localized heat to treat tumors by inducing localized tissue thermal damage. The present study aimed to evaluate the temperature evolution and spatial distribution, ablation size, and reproducibility of ablation zones in ex vivo liver, kidney, and lung using a commercial device, i.e., Dophi™ R150E RFA system (Surgnova, Beijing, China), and to compare the results with the manufacturer’s specifications. Optical fibers embedding arrays of fiber Bragg grating (FBG) sensors, characterized by 0.1 °C accuracy and 1.2 mm spatial resolution, were employed for thermometry during the procedures. Experiments were conducted for all the organs in two different configurations: single-electrode (200 W for 12 min) and double-electrode (200 W for 9 min). Results demonstrated consistent and reproducible ablation zones across all organ types, with variations in temperature distribution and ablation size influenced by tissue characteristics and RFA settings. Higher temperatures were achieved in the liver; conversely, the lung exhibited the smallest ablation zone and the lowest maximum temperatures. The study found that using two electrodes for 9 min produced larger, more rounded ablation areas compared to a single electrode for 12 min. Our findings support the efficacy of the RFA system and highlight the need for tailored RFA parameters based on organ type and tumor properties. This research provides insights into the characterization of RFA systems for optimizing RFA techniques and underscores the importance of accurate thermometry and precise procedural planning to enhance clinical outcomes.

## 1. Introduction

Radiofrequency ablation (RFA) is a medical procedure that employs localized heat generated by radiofrequency energy to treat specific medical conditions [1]. RFA induces the destruction of biological tissues using electricity from unmodulated sinusoidal waves, alternating current at an electromagnetic frequency that falls within the range characteristics of radio broadcasting signals, preferably around 500 kHz. This frequency is in the medium wave range (i.e., 300–3000 kHz), high enough to cause molecular frictional heating without stimulating neuromuscular reaction and electrolysis, and low enough (<20 MHz) to limit energy transmission without generating excessive radiation to tissues [1,2,3]. This electrical alternating current causes ionic agitation and resistive heating of the biological tissues [4].

To establish the necessary and correct current, the RFA system requires a closed loop comprised of an electrical generator, a needle electrode, a patient (a resistor), and large dispersive electrodes (or, in many cases, “grounding pads”) [5,6,7]. RFA procedures are generally performed by using needle electrodes with different diameters and lengths placed under image guidance (computed tomography, magnetic resonance imaging, ultrasound imaging) into the tumor to be ablated. RFA has shown promise in treating selected solid tumors [8], like liver [9,10,11,12], kidney [13,14,15,16], and lung [16,17,18,19] tumors.

RFA is a well-established thermal ablation technique, extensively validated in the literature for its efficacy and safety, as outlined by the CIRSE (Cardiovascular and Interventional Radiology Society of Europe) guidelines [20,21,22]. It enables the treatment of various tumors by delivering localized thermal energy through a needle electrode, which is inserted directly into the lesion under real-time image guidance, such as ultrasound (US), computed tomography (CT), or magnetic resonance imaging (MRI). The use of imaging ensures precise electrode placement, reducing the risk to surrounding healthy tissues and optimizing treatment outcomes [23]. RFA is particularly effective in managing hepatocellular carcinoma (HCC), colorectal liver metastases, as well as primary and secondary lung and renal tumors [24,25,26]. This technique offers a minimally invasive option for patients who are not surgical candidates, ensuring localized tumor control while maintaining a favorable safety profile [27,28].

Biologically, the effects of heat-related therapies involve multiple complex mechanisms and depend on factors such as the temperature and duration of heat exposure, as well as local considerations like organ perfusion, tissue density, and electrolyte concentration. Due to these intricate dependencies, understanding how heat interacts with tissue to induce cell death is crucial.

Cellular homeostasis can be maintained with a mild elevation of temperature to approximately 40 °C. As temperatures increase to the range of 42–45 °C, cells become more susceptible to damage by other agents, such as chemotherapy and radiation [29]. When temperatures escalate to 46 °C for over an hour, irreversible cellular damage occurs, highlighting the critical impact of prolonged exposure to elevated temperatures.

Furthermore, increasing the temperature by only several degrees to 50–52 °C for a shorter duration induces cytotoxicity, emphasizing the sensitivity of cells to even modest changes in thermal conditions. In the temperature range of 60–100 °C, an instantaneous induction of protein coagulation takes place, causing irreversible damage to key cytosolic and mitochondrial enzymes [30,31,32]. This information underscores the complexity of the relationship between temperature parameters and cellular responses in the context of heat-based therapeutic interventions [33,34]. Temperature exceeding 105 °C results in tissue boiling, vaporization, and carbonization, thereby compromising optimal ablation due to decreasing energy transmission [35]. Consequently, the primary objective becomes maintaining a temperature range of 50–100 °C throughout the entire target volume during ablation therapy. Achieving effective ablation is contingent upon minimizing heat loss, where the disparity between heat production and heat loss is termed heat efficacy [4].

While heat production correlates with the intensity and duration of the deposited RF energy, heat loss primarily stems from blood flow within adjacent vessels, known as the heat-sink effect [36]. This phenomenon is particularly affecting RFA, and it was demonstrated that minimal vascular flows (1 mL/min from the vessel at 5 mm distance from the electrode) cause evident heat sink effects in liver tissue [37]. As previously mentioned, effective RFA diminishes when tissues are heated beyond 100 °C, and the swift loss of heat at a distance from the electrode imposes limitations on the ablation size. This poses a challenge as the goal is to attain an ablation zone encompassing areas beyond the tumor margin to ensure the eradication of microscopic tumoral extensions, referred to as the ablation margin, ideally set at approximately 0.5–1.0 cm [6,9,10].

Among the advantages of RFA are its precision and targeting, its adaptability to various tissues, its ease of use, and the fact that it requires a minimal hospital stay. However, challenges and disadvantages persist, particularly with critical structures (e.g., bile duct, blood vessels), as their heat-sinking effects diminish the efficacy of the treatment [36] and with high temperatures that are difficult to control and can cause thermal damage to surrounding healthy structures. Therefore, investigating the thermal outcomes in tissue subjected to RFA and the attained ablation dimensions is fundamental to the final aim of characterizing the performance of RFA devices and toward the selection of the proper procedural settings.

This work focuses on the evaluation of the temperature distribution, ablation size, and reproducibility of ablation zones in three different ex vivo organs (liver, lung, and kidney) using a commercial device for RFA (Dophi™ R150E Radiofrequency Ablation System, by Surgnova Healthcare Technologies, Beijing, China) and compares the results obtained with those provided by the manufacturer.

To monitor the temperature variations occurring in ex vivo liver, kidney, and lung undergoing RFA, we employed fiber Bragg grating (FBG) sensors.

FBG sensors are characterized by immunity to electromagnetic fields, a small form factor, and good metrological characteristics (millimeter resolution, high accuracy, and fast response times) [38,39]. Additionally, they can be inscribed in arrays along the same optical fiber, enabling quasi-distributed measurements thanks to wavelength-division multiplexing obtained by varying the grating periods of the sensors along the fiber [40].

The ability to perform multipoint measurements with a single optical fiber and the other previously mentioned features are particularly interesting for assessing temperature variations during thermal treatments [38], where specific criteria must be met to achieve accurate thermometry (e.g., 1–2 °C accuracy, 1–2 mm spatial resolutions, and acquisition times in the range of seconds [41,42]). Hence, these sensors were selected for our analyses.

## 2. Materials and Methods

### 2.1. Experimental Setup

**Figure 1:**
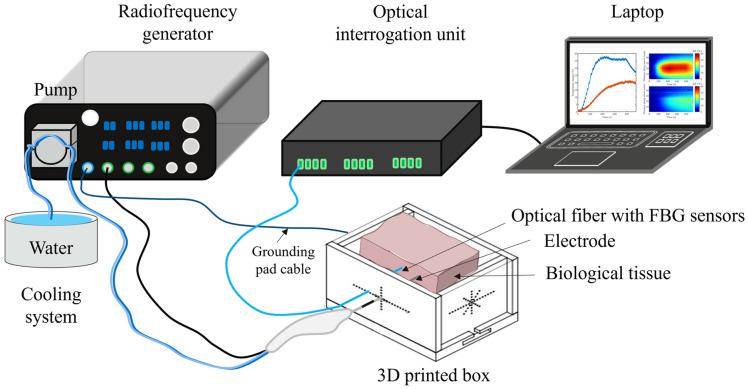
Experimental setup of RFA in liver, kidney, and lung. The experimental setup includes the RF generator connected to the water-cooled electrode(s) and the grounding pad; the biological tissue sample (bovine liver, kidney, or lung tissue); the optical interrogation unit connected to the optical fiber(s), embedding a chain of FBG sensors, and a laptop for displaying temperature evolution and distribution measured by the FBG sensors.

The RFA experimental setup comprises a custom-made box, an RFA system (Dophi™ R150E system (Surgnova, Beijing, China)) including a generator, electrodes, a cooling circuit, grounding pads, optical fibers equipped with fiber Bragg grating (FBG) sensors, an optical interrogator, and a computer for real-time temperature monitoring throughout the ablation process (Figure 1). The box was fabricated using a 3D printer, constructed from ABS material, and equipped with holes in the walls to allow the correct alignment of electrodes and sensors.

RFA was carried out on ex vivo liver, kidney, and lung. The fresh organs were procured from a local butcher, and each organ underwent distinct preparation before being employed in the experiment. Specifically, due to their substantial sizes, the lungs and liver needed to be cut into smaller pieces to fit within the box designated for the experiments. On the other hand, due to their reduced size, the kidneys required no treatment or cutting, as a single kidney perfectly fit inside the box. The primary focus during organ cutting was to provide sufficient space for the ablation process. To achieve this, reference to tables provided by the company was necessary to determine the expected ablation size, considering various experimental configurations associated with different ablation dimensions. Once the desired specimen was obtained, it was placed inside the 3D-printed box made of polymeric material above the grounding pad.

### 2.2. Thermometry Performed with FBGs

For the temperature distribution measurements deep within the tissue induced by RFA, up to three fibers containing an array of FBG sensors, coupled with the Micron Optics si255 interrogation unit (Micron Optics, Atlanta, GA, USA, 1 pm accuracy corresponding to 0.1 °C), were employed. Each fiber housed an array of 40 FBG sensors. The array was inscribed in the polyimide-coated single-mode fiber (SM1500(9/125)P, Fibercore, Ltd., Southampton, UK) through femtosecond laser point-by-point technology. The characteristics of each grating were the following: 1.15 mm grating length and 0.05 mm edge-to-edge distance between consecutive gratings. Hence, the spatial resolution was 1.2 mm, and the total sensing length, considering the 40 gratings for each array, was 48 mm. The spectral range of the interrogation unit was 1460–1620 nm. The FBG sensors were uniformly distributed in this region of the spectrum. Moreover, to guarantee a high dynamic range, i.e., >30 dB, the reflection coefficient of each FBG sensor was between 10% and 20% [39]. The FBG arrays were calibrated in a certified thermostatic dry-block calibrator (Giussani Quartz, operative field from −40 °C to 125 °C), providing a thermal sensitivity of (7.43 ± 0.01) × 10^−6^ °C^−1^ [38].

**Figure 2:**
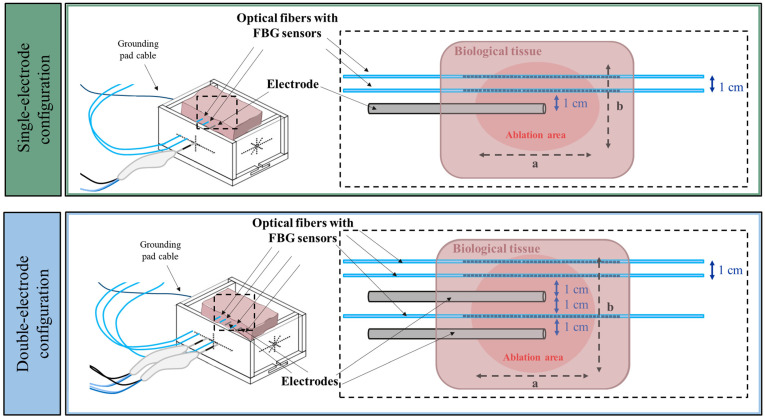
Placement of the electrodes and FBG sensors in the organs and definition of the ablation axes ‘a’ and ‘b’. On the left, the experimental setup is illustrated, while an enlargement of the position of the electrodes and optical fibers in the sample is shown on the right.

The optical fibers housing the FBGs were inserted in the organs with the help of a hollow needle, which was pulled out of the box before each RFA experiment, leaving the fiber in place. This operation was repeated for each required optical fiber: twice for the single-electrode setup since two arrays of FBG sensors were used, and three times for the double-electrode configuration since three arrays of FBG sensors were employed for thermometry within the tissue sample (Figure 2).

The upper part of the figure shows the single-electrode configuration, in which a single electrode is inserted into the tissue, and two fiber optics with FBGs sensors are inserted parallel to the electrode at a distance of 1 cm and 2 cm, respectively. In the double-electrode configuration (lower part of the figure), two electrodes are inserted into the tissue at a distance of 2 cm from each other. In addition, an optical fiber with FBGs sensors is inserted between the two electrodes, and two optical fibers with FBGs sensors are inserted parallel to the electrodes at a distance of 1 cm and 2 cm, respectively, from one of the electrodes. The configuration of the FBG arrays in the organs leads to a spatial resolution of 1.2 mm in the *x* direction and 1 cm in the *y* direction.

In order to quantify the ablation area, the ablation axes ‘a’ and ‘b’ have been defined. For each test, the initial organ temperature was measured with a K-type thermocouple (0.1 °C accuracy) and configured in a custom-made software developed in LabVIEW for displaying temperature variations measured by the FBGs. Temperature data are presented in two formats: the maximum temperature trends at distances of 1 cm and 2 cm from the single RF-electrode and between the two RF electrodes in the double-electrode configuration. These results are reported as (i) mean temperature values, calculated from three repeated tests, and (ii) 2D temperature evolution maps recorded along the sensors. The results of a single test randomly selected among the three repetitions have been reported for each organ. This choice is motivated by the fact that the RFA procedure is affected by power roll-off, i.e., by the continued interruption of the delivered current based on tissue properties (impedance) measured by the RF system during the procedure. For this reason, each test is different in terms of temperature trends.

### 2.3. RFA Settings

Based on the typical RFA procedures performed in clinical practice [20,21,22,27,28], we performed experiments with a single RF electrode and with two RF electrodes (Figure 2). Following the guide of the manufacturer, 17 G electrodes with an exposed tip of 2 cm were used for treating the soft tissues (liver, kidney, and lung) selected in our study. The following RFA settings were applied: (i) in the single-electrode configuration, 200 W for 12 min; (ii) in the double-electrode configuration, 200 W for 9 min. These settings were selected from the company datasheet and were indicated as suitable for both liver and lung. The RFA system was operated under the “auto mode”, which does not stop energy delivery after the tissue reaches a certain impedance but allows performing the procedures according to the chosen power and time. For each organ and each RFA setting, the experiments were repeated three times under identical conditions.

The electrodes are connected to an external pump, allowing water to circulate on the shaft to avoid tissue and electrode overheating. Thus, each electrode is equipped with a thermocouple that measures the water temperature.

### 2.4. Post-RFA Dimension Analysis

Once the RF generator was turned off, the electrode (or electrodes) and optical fibers could be extracted from the organ within the box, and the ablation size analysis could proceed. The organ was removed from the box, placed on an absorbent surface, and carefully cut along the electrode path. It was essential to track the electrode positions inside the organ and the insertion direction for accurate cutting.

The ablation axes (as depicted in Figure 2, marked as ‘a’ and ‘b’) were visually inspected and measured using a ruler. The interventional radiologists indicated the ablation margins for each experiment based on the visual inspection of the tissue color change induced by RFA. The visual tissue color changes induced by RFA correspond to alterations in vascularization, which can be detectable post-procedure using imaging modalities such as CT, US, or MRI [20,21,22]. The ablation axis ‘a’ is parallel to the antenna axis, and ‘b’ is perpendicular to the antenna axis.

### 2.5. Data Analysis Protocol

The results obtained in the different configurations were analyzed in terms of ablation size and attained temperature change. Tables were compiled containing data on the size of the ablated area, divided by setup (single/double-electrode configurations, power, time) and type of organ on which the experiment was performed. Three experiments per setup were considered, and once all data had been collected, the standard deviation and arithmetic mean of the length of the ‘a’ and ‘b’ axes were calculated from the acquired measurements. These results are reported as mean axis ± standard deviation and were compared with the data provided by the manufacturer.

From the mean values of the ablation axes, two indexes were computed: the sphericity index, calculated as b/a, and the (mean ablation size/power) × 100, which represents a metric of the mean ablation size per RF power unit.

## 3. Results

Results are organized as follows: first, the temperature trends and the maps showing the thermal evolution in time and space are reported for each organ; afterward, the ablation regions’ dimensions are reported.

### 3.1. Temperature Trends and Maps

#### 3.1.1. Liver

**Figure 3:**
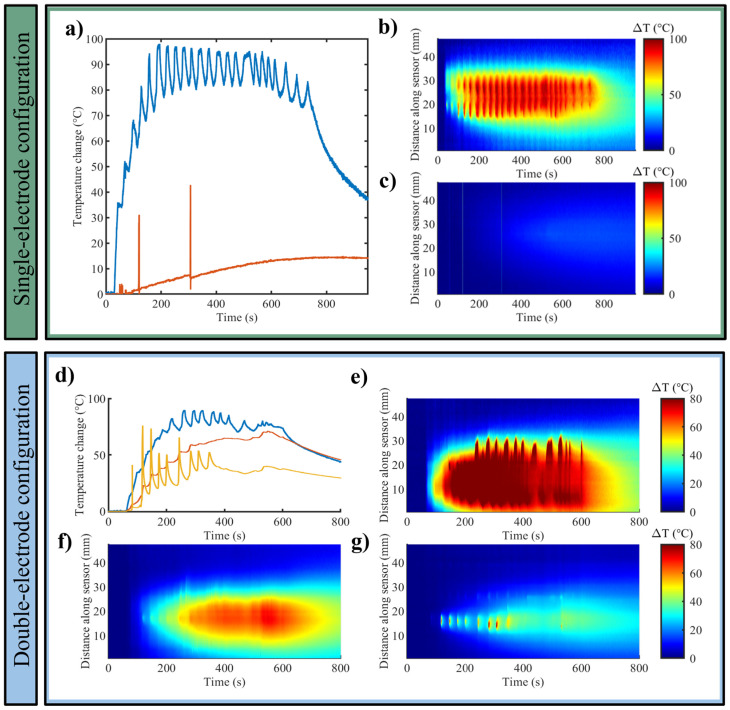
Bovine liver. Single-electrode configuration: (**a**) trends of maximum temperature variation at distances of 1 cm (blue) and 2 cm (red) from the RF electrode, and 2D temperature evolution maps along the sensor over time at a distance of (**b**) 1 cm and (**c**) 2 cm from the electrode. Double-electrode configuration: (**d**) trends of maximum temperature registered between the electrodes (blue), at 1 cm (red) and 2 cm (yellow) from the RF electrode; 2D temperature evolution maps along the sensor over time (**e**) between the two electrodes, at a distance of (**f**) 1 cm and (**g**) 2 cm from the electrode.

Figure 3 shows the results in terms of (i) the maximum temperature trends measured by each FBG array when one RF electrode, i.e., single-electrode configuration (Figure 3a) and two RF electrodes, i.e., double-electrode configuration (Figure 3d) were used for RFA in the liver; (ii) the temperature distributed along the FBG axes and evolving in time, in case of one RF electrode (Figure 3b,c) and two RF electrodes (Figure 3e–g). Results are reported for one of the three experiments for each setting.

For the 200 W—12 min setup, the highest temperature change reached at 1 cm from the electrode was approximately 98 °C, while the temperature change trend registered at 2 cm from the electrode was mainly lower than 15 °C, besides the presence of two spikes characterized by temperature changes >30 °C) (Figure 3a). In the case of the 200 W—9 min setup, in the double-electrode configuration, the maximum temperature change was found between the two electrodes, and it was approximately 90 °C. At the end of the heating procedure, the registered temperature changes were around 74 °C, 66 °C, and 38 °C between the electrodes and at 1 cm and 2 cm from the electrode, accordingly (Figure 3d).

In Figure 3b,c,e–g, the graphs, having on the *x*-axis the time (s) and on the *y*-axis the distance along the sensor (mm), depict the 2D thermal distribution for the different cases. It is observable that especially the sensors placed at a 1 cm distance from the electrode have registered temperature spikes that likely reflect the variation of the power emitted by the RF generator to counteract the high impedance of the sample undergoing RFA.

#### 3.1.2. Kidney

**Figure 4:**
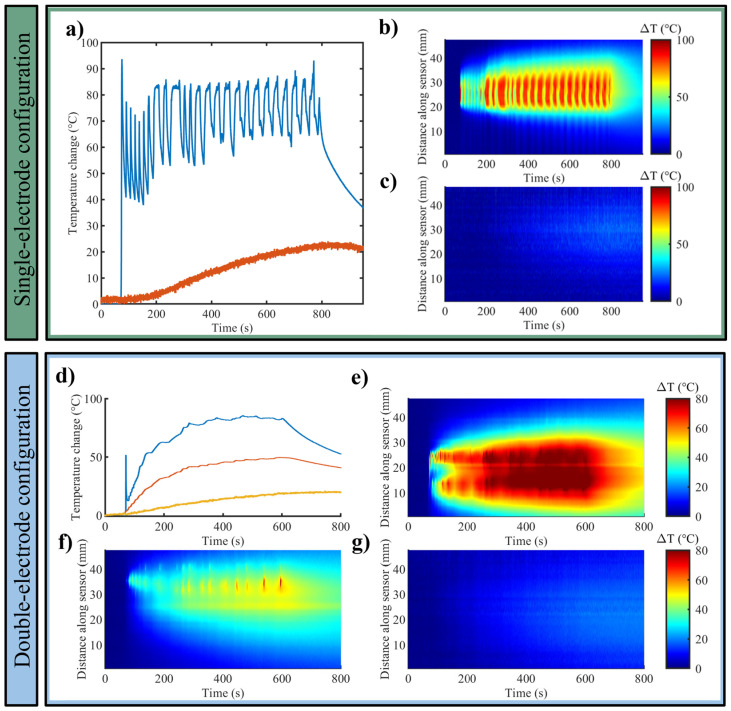
Bovine kidney. Single-electrode configuration: (**a**) trends of maximum temperature change at distances of 1 cm (blue) and 2 cm (red) from the RF electrode, and 2D temperature evolution maps along the sensor over time at a distance of (**b**) 1 cm and (**c**) 2 cm from the electrode. Double-electrode configuration: (**d**) trends of maximum temperature registered between the electrodes (blue), at 1 cm (red) and 2 cm (yellow) from the RF electrode; 2D temperature evolution maps along the sensor over time (**e**) between the two electrodes, at a distance of (**f**) 1 cm and (**g**) 2 cm from the electrode.

Figure 4 depicts the results concerning the profile of the maximum temperature change registered by each FBG array and the 2D thermal maps obtained for the RFA in the kidney (one exemplificative trial is shown out of the three experiments for each setting).

For the 200 W—12 min set up at 1 cm and 2 cm distances, the maximum values of temperature change were about 93 °C and 24 °C, respectively (Figure 4a). For the double-electrode configuration (200 W—9 min settings), the registered maximum values were 86 °C, 50 °C, and 20 °C between the electrodes, at 1 cm and 2 cm from the RF electrode, accordingly (Figure 4d).

Regarding the 2D thermal maps, in the case of the single-electrode configuration experiments, especially for the optical fiber sensor placed at 1 cm distance from the electrode (Figure 4b), multiple temperature spikes are visible. As previously mentioned, these spikes are likely attributable to the continuous changes in the power emitted by the generator to counteract the high impedance.

#### 3.1.3. Lung

**Figure 5:**
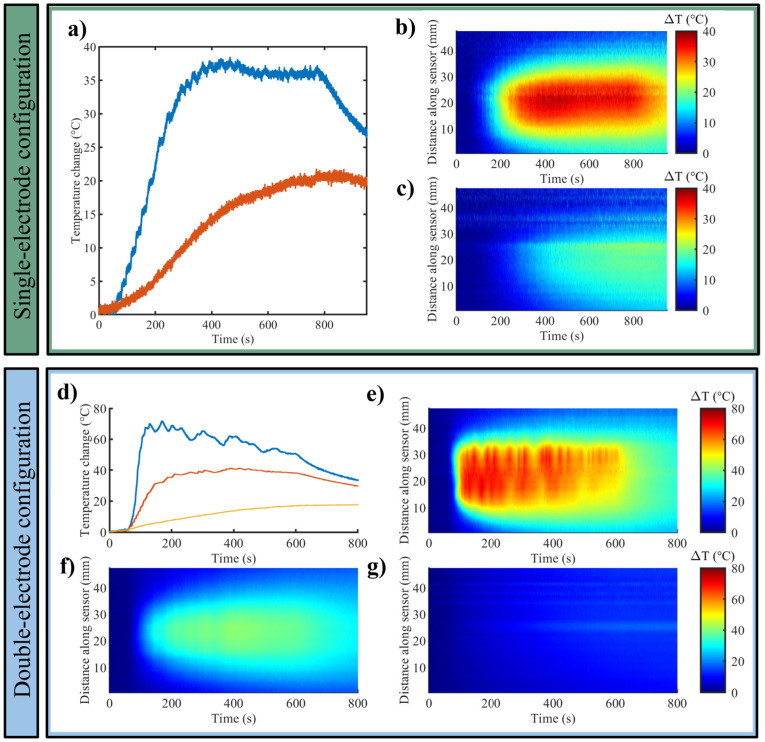
Bovine lung. The results of the single-electrode configuration experiments are shown in the upper panel: (**a**) trends of maximum temperature change at distances of 1 cm (blue) and 2 cm (red) from the electrode, and 2D temperature change maps along the sensor over time at a distance of (**b**) 1 cm and (**c**) 2 cm from the electrode. Double-electrode configuration (lower panel): (**d**) trends of maximum temperature registered between the electrodes (blue), at 1 cm (red) and 2 cm (yellow) from the RF electrode; 2D temperature evolution maps along the sensor over time (**e**) between the two electrodes, at a distance of (**f**) 1 cm and (**g**) 2 cm from the electrode.

As far as ex vivo lung tissues are concerned, the maximum temperature trends measured by each FBG array for the single-electrode configuration and double-electrode configuration are depicted in Figure 5a,d, correspondingly. Moreover, the temperature variations, distributed along the FBG axes and evolving in time, are reported in the case of single-electrode configuration (Figure 5b,c) and double-electrode configuration (Figure 5e–g). As also specified for the liver and kidney, the results are reported for one of the three experiments for each setting.

For the 200 W—12 min setup at 1 cm and 2 cm from the electrode (single-electrode configuration), the maximum temperature values reached were approximately 39 °C and 22 °C, respectively (Figure 5a). Concerning the 200 W—9 min setup (double-electrode configuration), between the electrodes and at 1 cm and 2 cm distances from the electrode, the attained maximum temperature changes were around 72 °C, 42 °C, and 18 °C (Figure 5d). It is worth highlighting that in the cases of kidney and liver tissues, the reported maximum values of temperature change attained during RFA treatments were approximately 1.3–1.4-fold higher compared with the maximum temperature change registered in lung tissue.

### 3.2. Post-RFA Analysis of the Ablation Region Dimension

#### 3.2.1. Liver

**Figure 6:**
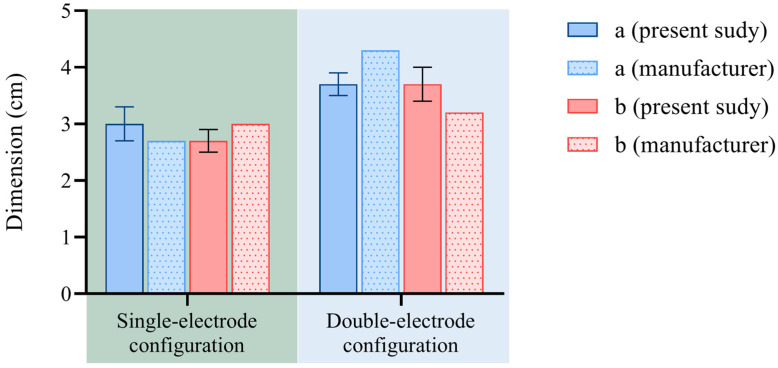
Bovine liver: ablation axes obtained from ex vivo experiments compared with the manufacturer’s data at different settings (i.e., 200 W for 12 min in the case of single-electrode configuration and 200 W for 9 min for the double-electrode configuration).

Figure 6 and Table 1 show the results from the ablation region dimensional analysis performed in this study on the liver and the comparison with data reported by the manufacturer of the RFA system.

The mean values of ‘a’ and ‘b’ are overall consistent with the data provided by the manufacturer. Regarding the standard deviation, in the single-electrode setup (i.e., with a power of 200 W and a duration of 12 min), its value is 0.3 mm for ‘a’ and 0.2 mm for ’b’; for the double-electrode configuration and, therefore with a power of 200 W and a duration of 9 min, the standard deviation is 0.2 mm for ‘a’ and 0.3 mm for ‘b’. The sphericity index close to 1 in both electrode configurations indicates that a spherical ablation has been achieved. The ablation size per power unit is 1.43 and 1.85 for the single-electrode setup and the double-electrode setup, respectively.

#### 3.2.2. Kidney

**Figure 7:**
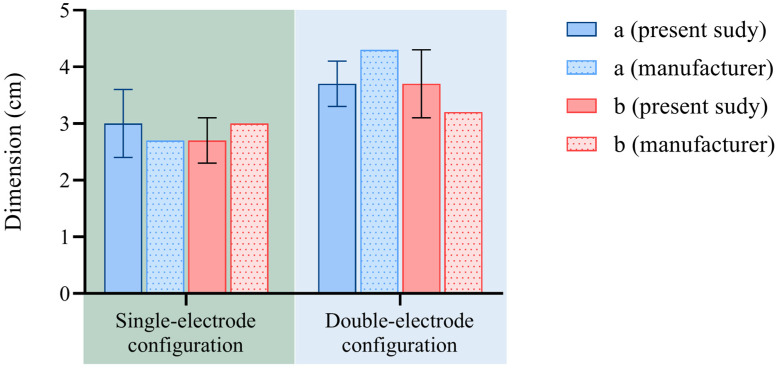
Bovine kidney: ablation axes obtained from ex vivo experiments compared with the company’s data at different settings (i.e., 200 W for 12 min in the case of single-electrode configuration and 200 W for 9 min for the double-electrode configuration).

Regarding the experimental evaluations performed on the kidney, Figure 7 shows the results from the size evaluation performed in the present study alongside the data provided by the manufacturer of the RFA system for the liver tissue. Table 2 lists the results and further information obtained from the ablation axes measurements.

In the case of the kidney, the company does not provide reference values for the RF settings analyzed. Thus, following the interventional radiologists’ indication regarding the clinical practice, we considered those used for the liver, given the similarity between the density and thermal properties of these two organs [43,44,45,46].

According to Figure 7, for 200 W—12 min, the measurements are comparable to the company’s data for the axis perpendicular to the electrode (b) with a standard deviation < 4 mm, while for 200 W—9 min, for ‘a’ the data are comparable, but for ‘b’ the standard deviation is 6 mm. The main causes of these differences can be the following: firstly, from an anatomical perspective, the veal kidneys contain structures known as renal calyces and pelvis, which are visible once the kidney is dissected. These calyces represent a part of the urinary tract through which urine is transported to the ureter. They can be likened to vessels that contribute to a heat sink effect, causing the ablation to be interrupted in these areas. Secondly, the small size of the organ and its structure consisting of lobes separated by deep grooves into which adipose tissue infiltrates pose a challenge in performing complete ablations in the interior portion of the organ without affecting its surface or the internal fat. As observed for the liver, the experiments performed in the kidney also provided a sphericity index close to 1 in both electrode configurations, thus resulting in spherical ablations. The ablation size per power unit (1.43 for the single-electrode setup and 1.85 for the double-electrode setup) is comparable to the one achieved for the liver.

#### 3.2.3. Lung

**Figure 8:**
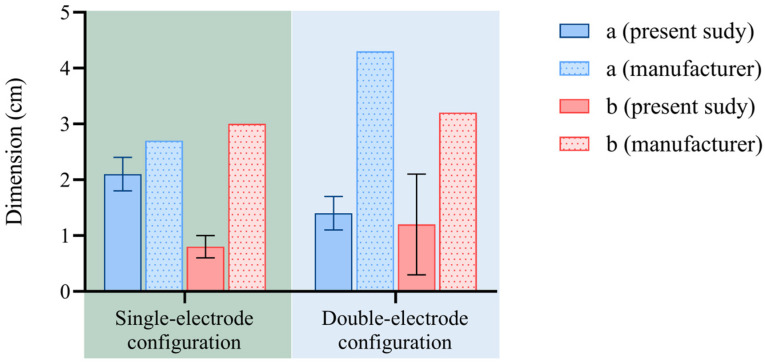
Bovine lung: ablation axes obtained from ex vivo experiments compared with the company’s data at different settings (i.e., 200 W for 12 min in the case of single-electrode configuration and 200 W for 9 min for the double-electrode configuration).

Concerning the lung, Figure 8 and Table 3 report the results in terms of ablation dimensions attained in this work and the comparison with the dimensions provided by the manufacturer of the RFA system, which are the same as the dimensions provided for the liver.

For lung tissue, the main differences in terms of ablation axes between the results of the present study and the values present in the manufacturer’s report can be observed (i.e., the maximum difference found for the ‘a’ axis with double-electrode configuration). Moreover, the standard deviation is 3 mm for ‘a’ and 2 mm for ‘b’ in the setting 200 W—12 min (single-electrode configuration), while it is 9 mm for ‘b’ for the 200 W—9 min setting (double-electrode configuration). The sphericity index resulted to be 0.38 in the setting 200 W—12 min (single-electrode configuration) and 0.86 in the setting 200 W—9 min (double-electrode configuration). The ablation size per power unit is smaller than 1 in both configurations.

## 4. Discussion and Conclusions

RFA has emerged as a valuable tool in the management of various solid tumors, offering a minimally invasive alternative to traditional surgical resection [11,12,47,48]. This technique utilizes localized heat generated by radiofrequency energy to induce tissue destruction, making it particularly suitable for tumors in organs like the liver, kidneys, and lungs [11,14,17,18,49]. Our study aimed to evaluate the temperature evolution, distribution, ablation size, and reproducibility of ablation zones in ex vivo liver, kidney, and lung specimens using a commercial RFA system (Dophi™ R150E system (Surgnova, Beijing, China)) and compare the attained results with those provided by the manufacturer.

The results of our experiments demonstrated that RFA produced consistent and reproducible ablation zones in all three organs investigated. FBG sensors allowed monitoring of the thermal effects in terms of evolution and spatial distribution of the temperature changes in the samples. The employed temperature measurement system based on FBG sensors, characterized by spatial resolution of 1.2 mm (in the *x* direction) and 0.1 °C accuracy, demonstrated to meet the requirements for adequate thermometry of the RFA procedure [41,42]. The temperature distribution analysis revealed distinct trends depending on the organ type and RFA settings. The spatial resolution of 1 cm provided by the FBG sensors in the *y* direction allows the capture of the temperature profiles in regions of the tissue which are of interest to the clinicians, i.e., within the electrodes and at 1 cm and 2 cm from the electrodes and is compatible with the size of the ablation margin. This information is ameliorative with respect to temperature data available during US-guided RFA in clinical practice [50].

The highest temperature values were achieved in the liver and kidney. In contrast, the lung, which is characterized by distinct thermophysical properties compared to the liver and kidney [32,44,45,46,51] and features air-filled bronchi that act as heat sinks, exhibited the smallest ablation zone and the lowest maximum temperatures.

Importantly, our study highlighted the impact of RFA settings on ablation outcomes. Different power levels and durations resulted in variations in ablation size and temperature distribution. For instance, higher power settings and longer durations generally led to larger ablation zones but also increased the risk of thermal damage to surrounding healthy structures. Conversely, lower power settings and shorter durations produced smaller ablation zones but minimized the risk of collateral damage.

In our study, we also compared the ablation areas obtained using a single electrode for 12 min and two electrodes for 9 min for each organ, aiming to achieve comparable ablation sizes as indicated by the manufacturer’s measurement guide. The dimensions of the ablated areas in the kidney and liver at both 12 and 9 min were similar, given that both are solid parenchymal organs. Moreover, RFA produced spherical ablations in both the liver and kidney, as indicated by the sphericity index close to 1 in the two RF settings used in this study. These findings justify the possibility of using the kidney for the RF settings provided for the liver, with comparable results.

In contrast, the lung, characterized by the presence of air, showed a smaller ablation area compared to the kidney and liver due to thermal insulation. In the lung, an oval-shaped ablation area was observed, especially at both 12 min with a single electrode, with a sphericity index equal to 0.38. Thus, the settings suggested for the liver do not produce the same thermal effect on the lung. Indeed, the index indicating ablation size per power unit is >1 for the liver and kidney and <1 for the lung, thus demonstrating that the same power units produce a smaller ablation in the lung with respect to the liver/kidney.

In general, the standard deviation along the ablation axes of the lung was consistently larger compared to that observed for the liver (Figure 6 and Figure 8), particularly along axis ‘b’, which runs perpendicular to the electrode’s path. This discrepancy may be attributed to the presence of multiple bronchi within the lung tissue, leading to a heat sink effect that causes reduced ablation consistency, especially when employing high-power settings [52]. This fact could also be related to the inner limitation of RFA, such as the heat sink effect and limited active deposition of energy into tissue. The fact that a significant standard deviation is observed in both single- and double-electrode configurations could be related to the limitation of the ablation zone due to the presence of anatomical structures.

Therefore, as expected, the lung exhibited different behavior compared to the kidney and liver [44,45,51,52].

According to our findings, the use of two electrodes for 9 min is preferable to a single electrode for 12 min, as it allows for a larger and rounded morphology ablation area in less time. A critical factor in achieving successful ablation and preventing residual tumor tissue or tumor recurrence is maintaining an adequate safety margin. Currently, there is no universally agreed-upon consensus regarding what constitutes an adequate safety distance; recent studies observed that the minimum safety margin for liver thermal ablation procedures should be in the range of 3 to 6 mm [53]. However, these data should be considered based on the anatomy and characteristics of the lesion being treated.

From a clinical perspective, our study has several implications. Firstly, it provides valuable information on the optimal RFA settings for achieving desired ablation outcomes in different organs. Clinicians can use this knowledge to tailor RFA parameters based on individual patients’ characteristics and tumor properties. Secondly, our results underscore the importance of meticulous planning and precise execution during RFA procedures to ensure optimal outcomes and minimize the risk of complications.

However, our study also has some limitations. Firstly, the experiments were conducted ex vivo using animal organs, which may not fully replicate the conditions encountered in vivo [54]. In particular, the heat-sink effect due to vasculature in living tissues is at the origin of the reduction of the ablation volume. Thus, the results of this work cannot be directly transferred into tumor treatment in patients but still provide useful guidance for the choice of the most suitable RFA settings. Physicians should then use monitoring devices to assess the evolution of the ablation region in real time. Additionally, the work focused on a specific commercial RFA system, and the results may not be generalizable to other RFA devices. Lastly, the accurate placement of the FBG sensors inside the organs should be guided through a noninvasive diagnostic system (e.g., US imaging) to detect anatomical structures, such as blood vessels and bronchi, which might affect the heat distribution inside the organs.

In conclusion, our study provides valuable insights into the FBG sensors-monitored temperature evolution and distribution, ablation size, and reproducibility of ablation zones in ex vivo liver, kidney, and lung specimens using a commercial RFA system. These findings contribute to the understanding of the factors influencing RFA outcomes and can inform clinical decision-making in the management of solid tumors. Further research in in vivo settings is warranted to validate our findings and optimize RFA techniques for improved patient outcomes.

## Figures and Tables

**Table 1:** Analysis of ablation axes produced by RFA in liver and comparison with data provided by the manufacturer.

		a [cm]	b [cm]	a − a_m_ [cm]	b − b_m_ [cm]	Sphericity Index, b/a	(Mean Ablation Size/Power) × 100 [cm/W]
Single electrode	RFA 200 W 12 min	3 ± 0.3	2.7 ± 0.2	0.3	−0.3	0.9	1.43
Two electrodes	RFA 200 W 9 min	3.7 ± 0.2	3.7 ± 0.3	−0.6	0.5	1	1.85

1 a_m_ = a provided by manufacturer; b_m_ = b provided by manufacturer.

**Table 2:** Analysis of ablation axes produced by RFA in kidney and comparison with data provided by the manufacturer.

		a [cm]	b [cm]	a − a_m_ [cm]	b − b_m_ [cm]	Sphericity Index	(Mean Ablation Size/Power) × 100 [cm/W]
Single electrode	RFA 200 W 12 min	3 ± 0.6	2.7 ± 0.4	0.3	−0.3	0.9	1.43
Two electrodes	RFA 200 W 9 min	3.7 ± 0.4	3.7 ± 0.6	−0.6	0.5	1	1.85

1 a_m_ = a provided by manufacturer; b_m_ = b provided by manufacturer.

**Table 3:** Analysis of ablation axes produced by RFA in lung and comparison with data provided by the manufacturer.

		a [cm]	b [cm]	a − a_m_ [cm]	b − b_m_ [cm]	Sphericity Index	(Mean Ablation Size/Power) × 100 [cm/W]
Single electrode	RFA 200 W 12 min	2.1 ± 0.3	0.8 ± 0.2	−0.6	−2.2	0.38	0.73
Two electrodes	RFA 200 W 9 min	1.4 ± 0.3	1.2 ± 0.9	−2.9	−2	0.86	0.65

1 a_m_ = a provided by manufacturer; b_m_ = b provided by manufacturer.

## Data Availability

The dataset is available on request from the authors.

## References

[1] Ni Y., Mulier S., Miao Y., Michel L., Marchal G. (2005). A Review of the General Aspects of Radiofrequency Ablation. Abdom. Imaging.

[2] Organ L.W. (2007). Electrophysiologic Principles of Radiofrequency Lesion Making. Appl. Neurophysiol..

[3] Hand J.W., Haar G. (1981). ter Heating Techniques in Hyperthermia. Br. J. Radiol..

[4] Tatli S., Tapan Ü., Morrison P.R., Silverman S.G. (2012). Radiofrequency Ablation: Technique and Clinical Applications. Diagn. Interv. Radiol..

[5] Strand N.H., Hagedorn J.M., Dunn T., Johnson B., Abd-Elsayed A., Covington S., Freeman J., Dawodu A., Maloney J. (2024). Advances in Radiofrequency Ablation: Mechanism of Action and Technology. Ann. Palliat. Med..

[6] Hong K., Georgiades C. (2010). Radiofrequency Ablation: Mechanism of Action and Devices. J. Vasc. Interv. Radiol..

[7] Boers T., Brink W., Bianchi L., Saccomandi P., van Hespen J., Wennemars G., Braak S., Versluis M., Manohar S. (2024). An Anthropomorphic Thyroid Phantom for Ultrasound-Guided Radiofrequency Ablation of Nodules. Med. Phys..

[8] Wood B.J., Ramkaransingh J.R., Fojo T., Walther M.M., Libutti S.K. (2002). Percutaneous Tumor Ablation with Radiofrequency. Cancer.

[9] Nakazawa T., Kokubu S., Shibuya A., Ono K., Watanabe M., Hidaka H., Tsuchihashi T., Saigenji K. (2007). Radiofrequency Ablation of Hepatocellular Carcinoma: Correlation Between Local Tumor Progression After Ablation and Ablative Margin. Am. J. Roentgenol..

[10] Kim J.W., Kim J.H., Won H.J., Shin Y.M., Yoon H.-K., Sung K.-B., Kim P.N. (2012). Hepatocellular Carcinomas 2–3 Cm in Diameter: Transarterial Chemoembolization plus Radiofrequency Ablation vs. Radiofrequency Ablation Alone. Eur. J. Radiol..

[11] Sutherland L.M., Williams J.A.R., Padbury R.T.A., Gotley D.C., Stokes B., Maddern G.J. (2006). Radiofrequency Ablation of Liver Tumors: A Systematic Review. Arch. Surg..

[12] Izzo F., Granata V., Grassi R., Fusco R., Palaia R., Delrio P., Carrafiello G., Azoulay D., Petrillo A., Curley S.A. (2019). Radiofrequency Ablation and Microwave Ablation in Liver Tumors: An Update. Oncologist.

[13] Hsu T.H.S., Fidler M.E., Gill I.S. (2000). Radiofrequency Ablation of the Kidney: Acute and Chronic Histology in Porcine Model. Urology.

[14] Salas N., Ramanathan R., Dummett S., Leveillee R.J. (2010). Results of Radiofrequency Kidney Tumor Ablation: Renal Function Preservation and Oncologic Efficacy. World J. Urol..

[15] Mahnken A.H., Günther R.W., Tacke J. (2004). Radiofrequency Ablation of Renal Tumors. Eur. Radiol..

[16] Brace C.L. (2009). Radiofrequency and Microwave Ablation of the Liver, Lung, Kidney, and Bone: What Are the Differences?. Curr. Probl. Diagn. Radiol..

[17] Zhu J.C., Yan T.D., Morris D.L. (2008). A Systematic Review of Radiofrequency Ablation for Lung Tumors. Ann. Surg. Oncol..

[18] Gadaleta C., Mattioli V., Colucci G., Cramarossa A., Lorusso V., Canniello E., Timurian A., Ranieri G., Fiorentini G., De Lena M. (2004). Radiofrequency Ablation of 40 Lung Neoplasms: Preliminary Results. Am. J. Roentgenol..

[19] Abtin F.G., Eradat J., Gutierrez A.J., Lee C., Fishbein M.C., Suh R.D. (2012). Radiofrequency Ablation of Lung Tumors: Imaging Features of the Postablation Zone. RadioGraphics.

[20] Ryan A., Byrne C., Pusceddu C., Buy X., Tsoumakidou G., Filippiadis D. (2022). CIRSE Standards of Practice on Thermal Ablation of Bone Tumours. Cardiovasc. Interv. Radiol..

[21] Crocetti L., de Baére T., Pereira P.L., Tarantino F.P. (2020). CIRSE Standards of Practice on Thermal Ablation of Liver Tumours. Cardiovasc. Interv. Radiol..

[22] Venturini M., Cariati M., Marra P., Masala S., Pereira P.L., Carrafiello G. (2020). CIRSE Standards of Practice on Thermal Ablation of Primary and Secondary Lung Tumours. Cardiovasc. Interv. Radiol..

[23] Clasen S., Pereira P.L. (2008). Magnetic Resonance Guidance for Radiofrequency Ablation of Liver Tumors. J. Magn. Reson. Imaging.

[24] Clasen S., Rempp H., Hoffmann R., Graf H., Pereira P.L., Claussen C.D. (2014). Image-Guided Radiofrequency Ablation of Hepatocellular Carcinoma (HCC): Is MR Guidance More Effective than CT Guidance?. Eur. J. Radiol..

[25] Minami Y., Kudo M. (2013). Radiofrequency Ablation of Liver Metastases from Colorectal Cancer: A Literature Review. Gut Liver.

[26] Ogan K., Jacomides L., Dolmatch B.L., Rivera F.J., Dellaria M.F., Josephs S.C., Cadeddu J.A. (2002). Percutaneous Radiofrequency Ablation of Renal Tumors: Technique, Limitations, and Morbidity. Urology.

[27] Grasso R.F., Bernetti C., Pacella G., Altomare C., Castiello G., Andresciani F., Sarli M., Zobel B.B., Faiella E. (2022). A Comparative Analysis of Thermal Ablation Techniques in the Treatment of Primary and Secondary Lung Tumors: A Single-Center Experience. Radiol. Med..

[28] Faiella E., Frauenfelder G., Santucci D., Luppi G., Zobel B.B., Grasso R.F. (2016). Percutaneous Radiofrequency Ablation of a Bleeding Pseudoaneurysm during CT-Guided Renal Cancer Treatment. A Case Report. Emerg. Radiol..

[29] Vujaskovic Z., Song C.W. (2004). Physiological Mechanisms Underlying Heat-Induced Radiosensitization. Int. J. Hyperth..

[30] Chu K.F., Dupuy D.E. (2014). Thermal Ablation of Tumours: Biological Mechanisms and Advances in Therapy. Nat. Rev. Cancer.

[31] Jaque D., Maestro L.M., del Rosal B., Haro-Gonzalez P., Benayas A., Plaza J.L., Rodríguez E.M., Solé J.G. (2014). Nanoparticles for Photothermal Therapies. Nanoscale.

[32] Bianchi L., Cavarzan F., Ciampitti L., Cremonesi M., Grilli F., Saccomandi P. (2022). Thermophysical and Mechanical Properties of Biological Tissues as a Function of Temperature: A Systematic Literature Review. Int. J. Hyperth..

[33] Goldberg S.N. (2001). Radiofrequency Tumor Ablation: Principles and Techniques. Eur. J. Ultrasound.

[34] Bianchi L., Baroni S., Paroni G., Violatto M.B., Moscatiello G.Y., Panini N., Russo L., Fiordaliso F., Colombo L., Diomede L. (2024). Thermal Effects and Biological Response of Breast and Pancreatic Cancer Cells Undergoing Gold Nanorod-Assisted Photothermal Therapy. J. Photochem. Photobiol. B Biol..

[35] Sonntag P.D., Hinshaw J.L., Lubner M.G., Brace C.L., Lee F.T. (2011). Thermal Ablation of Lung Tumors. Surg. Oncol. Clin..

[36] Ahmed M., Brace C.L., Lee F.T., Goldberg S.N. (2011). Principles of and Advances in Percutaneous Ablation. Radiology.

[37] Lehmann K.S., Poch F.G.M., Rieder C., Schenk A., Stroux A., Frericks B.B., Gemeinhardt O., Holmer C., Kreis M.E., Ritz J.P. (2016). Minimal Vascular Flows Cause Strong Heat Sink Effects in Hepatic Radiofrequency Ablation Ex Vivo. J. Hepato-Biliary-Pancreat. Sci..

[38] Namakshenas P., Bianchi L., Saccomandi P. (2023). Fiber Bragg Grating Sensors-Based Assessment of Laser Ablation on Pancreas at 808 and 1064 Nm Using a Diffusing Applicator: Experimental and Numerical Study. IEEE Sens. J..

[39] Korganbayev S., Orrico A., Bianchi L., Paloschi D., Wolf A., Dostovalov A., Saccomandi P. (2021). PID Controlling Approach Based on FBG Array Measurements for Laser Ablation of Pancreatic Tissues. IEEE Trans. Instrum. Meas..

[40] Kersey A.D., Davis M.A., Patrick H.J., LeBlanc M., Koo K.P., Askins C.G., Putnam M.A., Friebele E.J. (1997). Fiber Grating Sensors. J. Light. Technol..

[41] Frich L. (2006). Non-invasive Thermometry for Monitoring Hepatic Radiofrequency Ablation. Minim. Invasive Ther. Allied Technol..

[42] Lewis M.A., Staruch R.M., Chopra R. (2015). Thermometry and Ablation Monitoring with Ultrasound. Int. J. Hyperth..

[43] TISSUE DB IT’IS Foundation. https://itis.swiss/virtual-population/tissue-properties/database/.

[44] Mohammadi A., Bianchi L., Asadi S., Saccomandi P. (2021). Measurement of Ex Vivo Liver, Brain and Pancreas Thermal Properties as Function of Temperature. Sensors.

[45] Bianchi L., Fiorentini S., Gianella S., Gianotti S., Iadanza C., Asadi S., Saccomandi P. (2023). Measurement of Thermal Conductivity and Thermal Diffusivity of Porcine and Bovine Kidney Tissues at Supraphysiological Temperatures up to 93 °C. Sensors.

[46] Rossmann C., Haemmerich D. (2014). Review of Temperature Dependence of Thermal Properties, Dielectric Properties, and Perfusion of Biological Tissues at Hyperthermic and Ablation Temperatures. Crit. Rev.™ Biomed. Eng..

[47] Abdelsalam M.E., Awad A., Baiomy A., Irwin D., Karam J.A., Matin S.F., Sheth R.A., Habibollahi P., Odisio B.C., Lu T. (2023). Outcomes of Radiofrequency Ablation for Solitary T1a Renal Cell Carcinoma: A 20-Year Tertiary Cancer Center Experience. Cancers.

[48] Deng Q., He M., Fu C., Feng K., Ma K., Zhang L. (2022). Radiofrequency Ablation in the Treatment of Hepatocellular Carcinoma. Int. J. Hyperth..

[49] Han K., Kim J.H., Kim G.H., Kim J.H., Kim S.Y., Park S.H., Moon S., Kwon J.H., Kim G.M., Lee S.J. (2024). Radiofrequency Ablation of Subcapsular versus Nonsubcapsular Hepatocellular Carcinomas ≤ 3 Cm: Analysis of Long-Term Outcomes from Two Large-Volume Liver Centers. Eur. Radiol..

[50] Diehn F.E., Neeman Z., Hvizda J.L., Wood B.J. (2003). Remote Thermometry to Avoid Complications in Radiofrequency Ablation. J. Vasc. Interv. Radiol..

[51] Bianchi L., Bontempi M., De Simone S., Franceschet M., Saccomandi P. (2023). Temperature Dependence of Thermal Properties of Ex Vivo Porcine Heart and Lung in Hyperthermia and Ablative Temperature Ranges. Ann. Biomed. Eng..

[52] Miguel A.F., Delgado J.M.P.Q. (2012). Lungs as a Natural Porous Media: Architecture, Airflow Characteristics and Transport of Suspended Particles. Heat and Mass Transfer in Porous Media.

[53] Laimer G., Jaschke N., Schullian P., Putzer D., Eberle G., Solbiati M., Solbiati L., Goldberg S.N., Bale R. (2021). Volumetric Assessment of the Periablational Safety Margin after Thermal Ablation of Colorectal Liver Metastases. Eur. Radiol..

[54] Lu D.S.K., Raman S.S., Vodopich D.J., Wang M., Sayre J., Lassman C. (2002). Effect of Vessel Size on Creation of Hepatic Radiofrequency Lesions in Pigs. Am. J. Roentgenol..

